# EXAMINING THE RELATIONSHIPS BETWEEN RESILIENCE, CARDIOVASCULAR HEALTH, AND RACE AMONG US ADULTS

**DOI:** 10.1093/geroni/igae098.3296

**Published:** 2024-12-31

**Authors:** Briana Sprague, Kelly Mosesso

**Affiliations:** Indiana University Indianapolis, Indianapolis, Indiana, United States; Indiana University School of Medicine, Indianapolis, Indiana, United States

## Abstract

Modifiable risk and resilience factors explain up to 90% of cardiovascular disease risk. These may contribute to racial cardiovascular health (CVH) disparities, but there has been limited examination of the role of resilience on CVH and disparities. Our goal was to examine (1) which risk and resilience measures were associated with CVH and (2) whether there were differences in these measures by race among a sample of US adults. Aim 1’s sample included adults aged 34-84 from the MIDUS biomarker substudy (N = 1255). Aim 2’s sample comprised of adults aged 28-84 from the MIDUS parent study (N = 4702). The primary outcome of interest was CVH, operationalized as the AHA’s Life’s Essential 8 total score, behavior, and health subscores. The hypothesized resilience measures were psychological well-being (“PWB”; assessed with Ryff and Multidimensional Personality Scale), purpose in life, mindfulness, gratitude, and optimism (MPS subscales). Of those, greater PWB (Ryff), purpose in life, and optimism correlated with better Essential 8 total score and behavioral subscore (ps <.01). Higher PWB (MPS), as well as higher gratitude, were associated with better Essential 8 behavioral scores (p <.05). Of these, PWB (MPS), gratitude, and optimism were significantly differences by race, where Black adults had significantly greater values on all measures (p <.001). Implications for future research and practice will be discussed.

